# Editorial: Psychological frailty in aging: Lifespan trajectories and emerging risks

**DOI:** 10.3389/fpsyg.2022.998022

**Published:** 2022-09-09

**Authors:** Esperanza Navarro-Pardo, Elzbieta Bobrowicz-Campos, David Facal

**Affiliations:** ^1^Department of Developmental and Educational Psychology, University of Valencia, Valencia, Spain; ^2^Centre for Interdisciplinary Studies, University of Coimbra, Coimbra, Portugal; ^3^Centre for Psychological Research and Social Intervention at the ISCTE - University Institute of Lisbon, Lisbon, Portugal; ^4^Department of Developmental and Educational Psychology, University of Santiago de Compostela, Santiago de Compostela, Spain

**Keywords:** frailty, aging, lifespan trajectories, multidimensional and dynamic perspective, integrated healthcare

In the study of aging, “frailty” is the state that increases the individual's vulnerability to stress factors. In the context of biomedical sciences, the concept of frailty has been operationalized differently, and the most used is the frailty phenotype. However, different constructs have emerged in recent decades complementing the traditional one of physical frailty. From a biopsychosocial, gerontological outlook, multidimensional and dynamic perspectives that include physical, functional, cognitive, and psychosocial domains (e.g., cumulative deficit model), are currently more relevant (Rockwood and Mitnitski, 2007; Gobbens et al., 2010).

As people age and become frailer, their psychosocial circumstances seem to have a more direct impact on their health. For example, the concept of “cognitive frailty” includes the presence of physical frailty and mild cognitive impairment in the absence of dementia and/or disability (Facal et al.). “Social frailty” would be the risk of losing resources (e.g., social support, cohesive activities, and social participation) in the aging process to meet key social needs for human development. This lack of cognitive and social resources in old age can be accompanied by a withdrawal of vitality, as well as a loss in meaning of life and will-to-live (Bunt et al., 2017; Lozupone et al., 2020). Thus, “psychological frailty” (understood as a decrease in cognitive, social, and *transcendental* resources) would increase a person's vulnerability when exposed to stressful circumstances. In this way, there is no subordination between physical, psychological, and social areas, but rather these depend on different, interrelated developmental trajectories throughout the lifespan (Facal et al., 2019). Instead, what takes place is an interaction between socio-economic, familial, cognitive, and physiological factors present in aging (Navarro-Pardo et al., 2020). Similarly, the health crisis of COVID-19, as well as the psychosocial risks associated with the measures that governments around the world have adopted to stop the spread of the virus, could have a significant impact directly on physical health, mental health and frailty, as well as indirectly, as a consequence of restrictions in mobility, activity, and social and family relationships, isolation, increased difficulties in performing physical exercise, delay in access to services health and loss of autonomy, to benefit from other services that have moved to the online space (Lozupone et al., 2020; Maltese et al., 2020; Pelicioni et al., 2020; Holland et al., 2021; Garner et al., 2022).

Regarding the role of affective factors across lifespan, depression and frailty present positive bidirectional associations in old adults, share common risk factors and may share pathophysiologic pathways. In the longitudinal study by Cao et al., prefrail or frail participants showed higher risks of depressive symptoms before and after adjusting for sociodemographic and health confounders, compared with the robust participants. In the cross-sectional study by Yuan et al., depression was a significant mediator of the relationship between frailty and the self-perception of the aging process, and so a significant psychological predictor of frailty of old adults. In the study by Yao, poor childhood experiences (poor self-reported health in childhood, poor mothers' and/or fathers' mental health) were associated with higher odds of depression in later adulthood, stressing the role of lifespan development in old adults' mental health.

Another topic of relevance is the relationship between frailty and motor signs of aging. In this Research Topic, Lin et al. shave examined the longitudinal transitions in the phenotypes of old adults with impairments in mobility, cognitive functioning and both, showing the potential for reversibility of these impairments and identifying the predictors of convertibility of transitions between phenotypes in question.

In terms of biological aspects, aging is often accompanied by an increase in inflammation. Pothier et al. confirm significant links between inflammation (especially higher levels of C-reactive protein and interleukin-6) and frailty status. In our Research Topic three reviews directly point out to biomarkers able to predict or operativize the relation between biological and psychological processes in frailty (Carini et al.; Moyse et al.; Pothier et al.). In this respect, although the research about this topic has increased considerably in recent years, no clear common pathways have been demonstrated for both frailty and cognitive impairment. Changes in microbiota, plasma biomarkers such as IGF-1 and IGFBP2, metabolic factors including low levels of vitamin E alpha tocopherol, omega-6 and 3 and albumin (Facal et al.) and neuroinflamation (Moyse et al.), may be all involved in cognitive frailty. Carini et al. also propose the potential role of microRNAs such as iR-92a-3p and miR-532-5p as biomarkers of cognitive frailty.

Finally, and regarding intervention studies, two papers show the effectiveness of interventions based on physical activity and multicomponent in long-term care centers (Facal et al.) and home-based individual cognitive stimulation programs (Silva et al.). The home-based individual cognitive stimulation program proved to be a promising non-pharmacological alternative to address age-related cognitive changes, also having a positive effect on strengthening the relationship between the caregiver and the cared person. However, as this approach requires continuous involvement of caregivers, its success may depend on adequately mobilizing community responses. It is expected that these types of interventions addressing psychological frailty will increase progressively and intervention programs will obtain evidence of their effectiveness and efficiency in parallel with the aging of the population.

The comprehensive analysis of quantitative and qualitative evidence from different scientific areas, presented in this Research Topic, confirmed that frailty is an emerging health and societal challenge that requires a holistic and lifecycle-centered approach and involvement of actors from different sectors, including old adults themselves and their families. By adopting this new approach, the possibility arises of offering health and social care that respond to people's real needs and adjust to their circumstances, increasing the acceptability and commitment to the proposed treatment and contributing to its success. We hope that reflection prompted by our Research Topic will relevantly contribute to efforts to involve, empower, and encourage the population to be an active part of this process.

## Author contributions

All authors listed have made a substantial, direct, and intellectual contribution to the work and approved it for publication.

## Conflict of interest

The authors declare that the research was conducted in the absence of any commercial or financial relationships that could be construed as a potential conflict of interest.

## Publisher's note

All claims expressed in this article are solely those of the authors and do not necessarily represent those of their affiliated organizations, or those of the publisher, the editors and the reviewers. Any product that may be evaluated in this article, or claim that may be made by its manufacturer, is not guaranteed or endorsed by the publisher.

## References

[B1] Bunt S. Steverink N. Olthof J. van der Schans C. P. Hobbelen J. S. M. (2017). Social frailty in older adults: A scoping review. Eur. J. Ageing 14, 323–334. 10.1007/s10433-017-0414-7 28936141 PMC5587459

[B2] Facal D. Maseda A. Pereiro A. X. Gandoy-Crego M. Lorenzo-López L. Yanguas J.. (2019). Cognitive frailty: A conceptual systematic review and an operational proposal for future research. Maturitas 121, 48–56. 10.1016/j.maturitas.2018.12.006 30704565

[B3] Garner I. W. Varey S. Navarro-Pardo E. Marr C. Carol A. (2022). An observational cohort study of longitudinal impacts on frailty and well-being of COVID-19 lockdowns in older adults in England and Spain. Health Soc. Care in press. 10.1111/hsc.13735 PMC9545919 35089638

[B4] Gobbens R. J. Luijkx K. G. Wijnen-Sponselee M. Th Schols J. M. (2010). In search of an integral conceptual definition of frailty: opinions of experts. J. Am. Med. Dir. Assoc. 11, 338–343. 10.1016/j.jamda.2009.09.015 20511101

[B5] Holland C. Garner I. Simpson J. Eccles F. Navarro-Pardo E. Marr C.. (2021). Impacts of COVID-19 lockdowns on frailty and wellbeing in older people and those living with long-term conditions. Adv. Clin. Experi. Med. 30, 1111–1114. 10.17219/acem/144135 34821484

[B6] Lozupone M. La Montagna M. Di Gioia I. Sardone R. Resta E. Daniele A.. (2020). Social Frailty in the COVID-19 Pandemic Era. Front. Psychiatry 11:577113. 10.3389/fpsyt.2020.577113 33240129 PMC7669751

[B7] Maltese G. Corsonello A. Di Rosa M. Soraci L. Vitale C. Corica F.. (2020). Frailty and COVID-19: A systematic scoping review. J. Clin. Med. 9, 2106. 10.3390/jcm9072106 32635468 PMC7408623

[B8] Navarro-Pardo E. Facal D. Campos-Magdaleno M. Pereiro A. X. Juncos-Rabadán O. (2020). Prevalence of cognitive frailty, do psychosocial-related factors matter? Brain Sci. 10, 968. 10.3390/brainsci10120968 33322251 PMC7763872

[B9] Pelicioni P. H. S. Schulz-Moore J. S. Hale L. Canning C. G. Lord S. R. (2020). Lockdown during COVID-19 and the increase of frailty in people with neurological conditions. Front. Neurol. 11, 103389. 10.3389/fneur.2020.604299 33304316 PMC7701276

[B10] Rockwood K. Mitnitski A. B. (2007). Frailty in relation to the accumulation of deficits. J. Gerontol. 62, 722–727. 10.1093/gerona/62.7.722 17634318

